# A survey dataset on the perception of public-sector corruption in Mauritius and a framework analysis of corruption court cases

**DOI:** 10.1016/j.dib.2020.106057

**Published:** 2020-07-22

**Authors:** Sheilendra Peerthum, Rajendra Parsad Gunputh, Takesh Luckho

**Affiliations:** a Open University of Mauritius, Reduit, Mauritius; b University of Mauritius, Reduit, Mauritius; c Open University of Mauritius, Reduit, Mauritius

**Keywords:** Content analysis, Corruption, Independent commission against corruption (icac), Mauritius, Prevention of Corruption Act 2002

## Abstract

This data article provides research data on the perception of public-sector corruption in Mauritius, captured using a survey questionnaire as well as a thematic framework for the analysis of corruption court cases prosecuted by the national anti-corruption body, namely, the Independent Commission against Corruption (ICAC). A sequential mixed methods design was adopted. First, a quantitative approach was used, whereby a questionnaire was developed and administered on 600 persons to gauge public opinion on the perceived seriousness of the phenomenon of public-sector corruption in the country while at the same time recording citizens’ perceived effectiveness of the ICAC in its fight against public corruption in Mauritius. Second, to cross-validate the quantitative findings on the perceived effectiveness of the ICAC in fulfilling its mandate as an anti-corruption agency, a content analysis of court judgments prosecuted by ICAC from 2002 to 2018, was conducted to find out whether or not the respondents’ perceptions of the prosecutorial efficacy of the ICAC was justified. The questionnaire, the raw dataset as well as the analysis of court judgments can be downloaded from the Mendeley Data repository (https://data.mendeley.com/datasets/h8k5ghvhgh/1). The data may be reused for similar research in other parts of the world.

The research findings have been published in *The International Journal of Law, Crime, and Justice.*

Specifications table

**Table:** 

Subject	Social Sciences
Specific subject area	Public Policy; Corruption Law; Political Science
Type of data	Tables
	Charts
	Graphs/Figures
How data were acquired	1. A survey questionnaire. 2. Content Analysis of court judgments
	The questionnaire is available at: https://data.mendeley.com/datasets/h8k5ghvhgh/1
Data format	1. Survey data: Raw and Analysed 2. Thematic analysis of Court Judgments: Raw, Filtered, and Analysed
Parameters for data collection	1. The study population consisted of 893,518 voters for Mauritius (excluding Rodrigues island)) registered with the Electoral Commission of Mauritius, as of August 2018. A proportionate random sample of 600 persons was drawn from the Electoral Commission's register of registered voters of Mauritius. The recommended sample size for such type of analysis, according to Saunders, Lewis, and Thornhill, [1], would be a minimum of 384 (at the 95% confidence level). The current study employed a sample size of 600, which is more than adequate to constitute a representative sample of the population 2. A total of 94 judgments involving 120 persons were examined and analysed to determine, first the prosecutorial efficacy of the ICAC, and second the corruption typology reflected by the different types of corruption which were prevalent in the public sector. Only corruption cases prosecuted under the Prevention of Corruption Act 2002 (PoCA) were considered in this research.
Description of data collection	1. A questionnaire was administered by two research assistants from 3 September 2018 to 12 April 2019. Data was collected using a five-point Likert scale. Of the 600 identified voters on the electoral list, only 398 persons could be contacted. Two persons opted out while the data-collection exercise was ongoing, so that at the end of the day, a total of 396 valid responses, constituting a 66% response rate, was obtained and analysed. 2. Outcomes of the court judgments, which were available on the website of the Supreme Court of Mauritius were filtered, organised thematically, coded and analysed, both descriptively and inferentially. A dummy variable approach was used for the inferential analysis.
Data source location	Institution: The Open University of Mauritius- corresponding author: Sheilendra Peerthum. Email: peerthum@intnet.mu
	City/Town/Region: Réduit, Moka
	Country: Mauritius
	Latitude and longitude for collected samples/data: 20.2°S 57.5°E
	Primary data sources: Website of the Supreme Court of Mauritius: https://supremecourt.govmu.org/SitePages/HomePage.aspx
Data accessibility	Data are hosted on a public repository
	Repository name: Mendeley Data
	Data identification number: http://dx.doi.org/10.17632/h8k5ghvhgh.1
	and
	Direct URL to data: https://data.mendeley.com/datasets/h8k5ghvhgh/1
	Data identification number: http://dx.doi.org/10.17632/8k24gm3g3n.1
	and
	Direct URL to data: https://data.mendeley.com/datasets/8k24gm3g3n/1
Related research article	Peerthum, S., Gunputh, R. P., Luckho, T. Assessing the effectiveness of the fight against public-sector corruption in Mauritius: Perception v reality. International Journal of Law, Crime and Justice.
	https://doi.org/10.1016/j.ijlcj.2020.100419[2]

## Value of the data

• The survey questionnaire captures novel data on citizens’ perception of public corruption in Mauritius. The data can be used by both researchers and policy-makers to further analyse the relationship between citizens’ perception of corruption, and its corresponding determinants on other variables, in Mauritius and other countries. The data can as well be used for cross-country analysis.
• The thematic analysis of court judgments on public corruption cases in Mauritius is unique and provides an indication of the prosecutorial efficacy of the ICAC, which is a statutory anticorruption agency mandated to fight corruption. This method can be replicated in other jurisdictions towards the development of a generalised empirical typology of corruption in the public sector.
• The findings can be utilised by policymakers towards amending specific provisions of the current anti-corruption legislation.
• The findings may be used to suggest alternative measures for addressing public corruption, other than relying solely on the ICAC.
• The findings may as well serve to improve the efficiency of corruption fighting agencies.
• The research design and data analysis process might be reused by researchers in other countries, with a similar anti-corruption framework, for similar/comparative research purposes.

## 1 Data description

Two datasets are provided; the first dataset consists of the survey questionnaire as well as the coded responses.

## 2 Survey questionnaire

The survey questionnaire was used to capture respondents’ perception of corruption in the public sector of Mauritius and their beliefs on the effectiveness of the ICAC in fighting public corruption. The questionnaires contained an introduction informing participants of the aim of the research since respondents had to be apprised of the purpose and context of the exercise. Ethical issues, in particular, the need for participants to give their honest answers and the possibility of opting out at any point in time during the exercise if they felt morally uncomfortable or inconvenienced were also addressed. Questions were divided into two parts: In part A of the questionnaire, the data which was collected pertained to the demographic profile of respondents while part B attempted to measure the perception of corruption in the public sector in Mauritius. The first four opening questions introduced the topic of public-sector corruption by defining the scope of the public sector of Mauritius and captured information on the perceived seriousness of the problem of corruption and the extent to which the interviewees believed corruption was present in the public sector of Mauritius as well as their projections on the spread of corruption in the near future. The remaining five questions consisted of indicator variables, used to measure the effectiveness of the anti-corruption agency in Mauritius, namely the Independent Commission against Corruption (ICAC). A 5-point Likert scale was used to capture the responses obtained at Part B. Table 1 contains the list of questions, together with their main descriptive statistics. Provision was made during the interview for situations where the respondents were not able to answer a particular question. In such a case, the response was recorded as *“No Answer”* and coded as “99″ - as suggested by Carver and Nash [3]. The latter observations were excluded in the calculation of the mean and standard deviation.

**Table 1:** Descriptive Statistics.

Code	*Interview Questions (variables)*	*N*	*Minimum*	*Maximum*	*Mean*	*Std. Deviation*
Q7	According to you, how serious is the issue of corruption in Mauritius?	396	2	5	4.07	0.845
Q8	In your opinion, to what extent is corruption present in the public sector?	396	1	99	3.88	0.879
Q9	In your opinion, compared to last year, how has the level of corruption evolved?	396	1	99	3.90	0.748
Q10	How do you expect the level of corruption to evolve in the next three years	396	1	99	3.97	0.860
Q11	ICAC cannot be trusted to keep Mauritius corruption-free	396	1	5	2.18	0.780
Q12	ICAC cannot be trusted as it is not independent from political control.	396	1	4	2.27	0.840
Q13	The appointment of the Director of ICAC by the Prime Minister, rather than by an independent institution, means that ICAC is accountable towards the Prime Minister.	396	1	5	3.64	0.948
Q14	ICAC cannot be trusted as some of its investigations are politically motivated and directed towards political opponents.	396	1	4	2.27	0.872
Q15	ICAC cannot be trusted as it makes an abuse of its investigation powers by arresting people unnecessarily	396	1	5	2.42	0.901
Q16	In general, ICAC has not been successful in prosecuting corruption offences against public officials	396	1	5	2.43	0.771

**Fig. 1:**
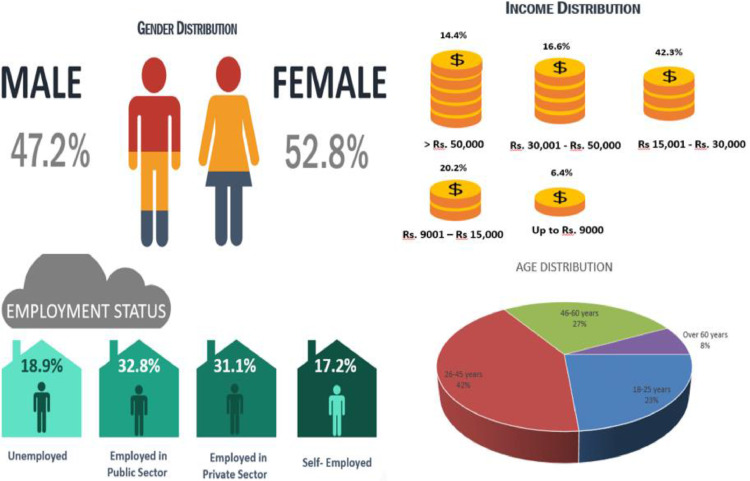
Demographic Profile of Respondents.

Fig. 1 summaries the main demographic characteristics of respondents who participated in the survey.

## 3 Coded responses

The survey responses which were already coded in the questionnaire were entered on a SPSS table. (‘Survey data –SPSS’ file). The variables have been defined and entered in the variable view tab using the same coding scheme of the survey questionnaire.

An Excel spreadsheet version containing the same data in CSV format is also available.(‘Survey data-EXCEL’ file)

The second dataset consists of a spreadsheet with the coded data used towards the inferential analysis, which has been extracted from a content analysis of 94 court judgments concerning 120 persons prosecuted by the ICAC in connection with suspected corruption offences (‘Data for inferential analysis’ file). The outcome of each case, which is the dependant variable, has been analysed against the different forms of corruption (TYPE) associated with each case; the places where the acts occurred (SECTOR) and the status of the accused party (STATUS).

Description of the coding scheme used in the ‘Data for inferential analysis’ spreadsheet

• The column ‘*Accused ID* ’ lists all the persons who were prosecuted by the ICAC for corruption offences, under the Prevention of Corruption Act 2002. To preserve anonymity, the accused parties have been given an alpha-numerical classification by substituting their names with the first alphabet of their surnames, followed by a number.
• The column *‘OUTCO*ME’ refers to the outcome of the prosecution, i.e. whether the case resulted in a dismissal (coded as 0) or a conviction (coded as 1). ‘OUTCOME’ is the dependant variable.
• The different types of corruption have been divided into two overarching categories for analysis purposes. *‘Bribery’* (coded as 2) refers to acts of corruption involving exchange of bribes; this includes receipt of a bribe, offer of a bribe and solicitation of a bribe. Non-bribery offences have been grouped as ‘*Misconduct in office’* (coded as 2) which comprise three activities, namely abuse of discretion, conflict of interest and violation of regulatory laws. ‘*TYPE’* is an independent variable.
• The column ‘SECTOR’ concerns the three sectors where the corrupt acts occurred and were grouped into three categories reflecting the public sector, coded as 1 for *“Central Government”*, 2 for *“Local Government”* and 3 for *“Statutory Body”*. ‘*SECTOR’* is an independent variable

Since there was only one case of corruption prosecuted in the private sector, it was excluded from our analysis.
The status of accused parties (STATUS) is also a dependant variable and has been classified on three levels as follows:
L1: *Operatives and junior officials*, coded as 1
L2: *Supervisory officials and middle-managers*, coded as 2, and
L3: *Top managers (heads of organisations and policymakers),* coded as 3

A summary statistics of the data is available in Table 2.

**Table 2:** Data Summary – Regression Analysis.

Variable	Obs*	Mean	Std. Dev.	Min	Max
OUTCOME	119	0.3697479	0.4847775	0	1
TYPE	119	1.596639	0.4926464	1	2
SECTOR	119	2.176471	1.571176	1	3
STATUS	119	2.806723	0.8465014	1	3

⁎ *One observation concerning a corruption case in the private sector has been excluded*.

## 4 Experimental design, materials and methods

The data have been processed and analysed in the context of a study which sought to answer the following two research questions:

  How do citizens of Mauritius assess the effectiveness of their national anti-corruption agency in fighting public-sector corruption?
  How are alleged corruption offences by public officials, under the Prevention of Corruption Act 2002 (PoCA), resolved by the courts of law?

Quantitative data, relating to attitudes, opinions, and beliefs are best captured through surveys [4]. According to Richards [5], *“surveys can provide an efficient means to ascertain public perceptions of corruption, and to understand the level and consequences of moral attitudes towards corrupt behaviour”*. A survey questionnaire was administered to capture the opinions of a representative sample of the Mauritian population aged 18 years and above, to address the first research question

The questionnaire was initially tested on 20 respondents and modified in the light of responses received. The revised questionnaire was then tested for reliability and validity. Since all the population is not fully conversant with the English language, a native version of the questionnaire in Mauritian Creole language was also drafted. The English version of the questionnaire is available at Mendeley data repository: https://data.mendeley.com/datasets/h8k5ghvhgh/1

The survey was carried held from the 3rd of September 2018 to the 12th of April 2019. Owing to possible interviewer bias, the authors refrained from interacting with the respondents and instead, two research assistants, one male and one female interviewer were recruited and trained to administer the questionnaires. During the seven months, the corresponding author supervised the conduct of the survey exercise and offered advice and guidance to the two research assistants as to how best to deal with the participants in order to gather their genuine and unbiased responses.

Of the 600 identified persons on the electoral list, only 398 replied positively to the invitation to participate in the survey. The remaining people, either, were not available during the period of the study or were simply not interested in the exercise. Before the start of the survey, the potential interviewees were informed that the information to be collected was in connection with research on the subject-matter of public corruption and that their permission was being sought to gather their honest opinions. Furthermore, they were given the assurance that their anonymity would be preserved and that they could discontinue the interview at any stage of the process if ever they felt that they could not proceed further. Only two persons opted out during the process and decided not to continue with the exercise. Therefore, at the end of the day, a total of 396 valid responses constituting a 66% response rate were received. The dataset of the responses is available in the data repository.

Since we used a mixed-methods design, we further investigated into a specific variable relating to the prosecutorial efficacy of the ICAC (Q16) by means of a ‘content analysis’ of judgments or outcomes of corruption cases. Court judgments have, therefore, been explored and analysed to answer the second research question. The objective of this method was to corroborate the quantitative findings of the research – in respect of respondents’ perception of the efficacy of the ICAC in prosecuting public officials with qualitative data, gathered through a study of the outcome of court judgments with a view to finding out the extent to which perceptions reflect the reality as measured by the success rate of the prosecutions.

The output approach to analyse the performance of ACAs [6] was used to examine the performance of the anticorruption agency of Mauritius by determining the percentage of convictions secured by the ICAC out of the total number of cases investigated and prosecuted. In this study, only corruption cases involving public officials prosecuted under the Prevention of Corruption Act 2002 (PoCA) have been analysed. Cases concerning money laundering and related offences prosecuted, under the Financial Intelligence and Anti-Money Laundering Act (FIAMLA) have been excluded. It should be noted that a few judgments were unavailable as these were not posted on the website of the Supreme Court. A total of ninety-four judgments (including 28, which were delivered by the Supreme Court upon appeal) were available as of 22 October 2018 and these documents, though being secondary sources of data, constitute primary sources of law, since court judgments, in the Mauritian judicial system, are considered as case laws.

Since the data were manageable, all the ninety-four cases were analysed rather than selecting a sample, so that all the inherent problems associated with sampling bias were eliminated. The sampling frame for this study, therefore, consisted of all the outcomes of these court cases, in the form of court judgments. The research method consisted of the following four steps:

1. Retrieving all available court judgments in respect of corruption offences, which allegedlyoccurred in the public sector of Mauritius, and which were prosecuted by ICAC since 2002.

2. Differentiating the judgements into two distinct sections, namely cases that were dismissed and those that resulted in a conviction.

3. Appraising data contained in the judgments to understand the *ratio decidendi* or the legal principle underlying the decision of the court.

4. Reducing the data to manageable proportions to look for patterns to understand better the behaviour of public officials and the multi-faceted aspects of public-sector corruption.

5. Synthesising and organising the resulting information to assess the prosecutorial efficacy of the ICAC.

Finally, an analytical framework, comprising only convicted persons, was drawn in line with Adam's Graycar's (2015) TASP(Types-Activities-Sectors-Places) framework for analysis of corrupt events [7]. This framework was slightly modified in our analysis by substituting ‘Place’ by ‘Perpetrator’ as the status of the offender is considered as a more determining element than the place where the alleged corrupt act occurred, given the context of the study. The TASP framework which also used for drawing an empirical typology of public corruption can be downloaded from the Mendeley data repository (http://dx.doi.org/10.17632/8k24gm3g3n.1#file-eb8d8408-c582–4066–8283–8f66ff104829)

## 5 Ethics statement

The authors declare that they have observed all ethical requirements for publication in *Data in Brief.*

## Declaration of Competing Interest

The authors declare that they have no known competing financial interests or personal relationships which have, or could be perceived to have, influenced the work reported in this article.
